# P71 Co-resistance network identifies antibiotic hub signals for empirical *Pseudomonas aeruginosa* wound therapy

**DOI:** 10.1093/jacamr/dlaf230.078

**Published:** 2025-12-04

**Authors:** Md Ebrahim Molla, Shahanaz Pervin, Zakir Hossain Habib, Shirin Tahmina, Quazi Ahmed Zaki

**Affiliations:** Khulna Medical College Hospital, Khulna, Bangladesh; Khulna Medical College, Khulna, Bangladesh; Institute of Epidemiology, Disease Control and Research (IEDCR), Dhaka, Bangladesh; Institute of Epidemiology, Disease Control and Research (IEDCR), Dhaka, Bangladesh; Institute of Epidemiology, Disease Control and Research (IEDCR), Dhaka, Bangladesh

## Abstract

**Background:**

*Pseudomonas aeruginosa* is a significant pathogen of wound infections with a high propensity for MDR, making it a challenging organism to treat. Although resistance rates of individual antibiotics are usually described, the relationships between resistances of various drug classes and co-occurrences of resistances are less understood, specifically in low- and middle-income countries. We assessed both resistance prevalence and co-resistance structure in a tertiary-care setting in Bangladesh to inform empirical therapy and stewardship.

**Objectives:**

To quantify resistance and MDR prevalence, map statistically robust co-resistance and hub antibiotics, and translate findings into pragmatic empirical stewardship signals.

**Methods:**

We conducted a retrospective cross-sectional study of 113 non-duplicate *P. aeruginosa* wound isolates at Khulna Medical College Hospital, Bangladesh (Aug 2018–Dec 2022). Susceptibility was tested using Kirby–Bauer disc diffusion interpreted per CLSI. Binary resistance to eight antipseudomonal antibiotics was correlated pairwise (Spearman’s ρ) with bootstrap 95% CIs; multiplicity was controlled with Benjamini–Hochberg FDR (q<0.05). A co-resistance network linked **antibiotic** pairs with significant |ρ|≥0.30; degree and betweenness centrality defined hubs.

**Results:**

Overall, 69.0% (78/113) of isolates were MDR. Resistance was highest to third- and fourth-generation cephalosporins: ceftazidime 85.0% and cefepime 81.4%. Resistance was lower for imipenem 23.9%, piperacillin/tazobactam 34.5% and amikacin 38.1%. Strong within-class co-resistance was observed for cefepime–ceftazidime (ρ=0.63; q<0.001) and amikacin–gentamicin (ρ=0.61; q<0.001). Cross-class links included ciprofloxacin–imipenem (ρ=0.48; q<0.001) and ciprofloxacin–piperacillin–tazobactam (ρ=0.45; q<0.001); moderate correlations were seen for ciprofloxacin–cefepime (ρ=0.33) and ceftazidime–piperacillin–tazobactam (ρ=0.31). The network was non-random (permutation *P<*0.001) with moderate clustering. Ciprofloxacin was the dominant hub (degree 6; betweenness 0.42), followed by gentamicin (degree 5; betweenness 0.31) and imipenem (degree 4; betweenness 0.28). Clinically, 89.2% of ciprofloxacin-resistant isolates were MDR, reinforcing the risk of empirical ciprofloxacin monotherapy. Conversely, 41.6% of isolates remained jointly susceptible to amikacin plus piperacillin–tazobactam, consistent with these agents’ peripheral network positions.

**Conclusions:**

In this high-burden setting, MDR *P. aeruginosa* is common, and resistance to cephalosporins is very high. A correlation-driven network highlights ciprofloxacin, imipenem and gentamicin as connectivity hubs that co-fail with multiple partners, whereas amikacin and piperacillin–tazobactam are more peripheral. Two stewardship-ready signals emerge: 1) avoid hub antibiotics for empirical monotherapy when severe wound infection is suspected, and 2) consider peripheral combinations such as amikacin plus piperacillin–tazobactam pending culture. Prospective, multi-centre validation with molecular profiling is warranted to confirm mechanisms and test the outcome impact of network-guided prescribing.

